# Comparison of Unilateral versus Bilateral Instrumented Transforaminal Lumbar Interbody Fusion in Lumbar Degenerative Diseases: A Minimum of 5-Year Follow-Up

**DOI:** 10.3390/medicina59111898

**Published:** 2023-10-26

**Authors:** Sung Cheol Park, Jae Seong Bae, Seon Ok Jung, Kyeong-Hoon Sung, Hoon-Jae Chung

**Affiliations:** 1 Department of Orthopedic Surgery, Bumin Hospital Seoul, Seoul 07590, Republic of Korea; neoz0708@gmail.com; 2 Department of Spine Surgery, Seoul 21st Century Spine Hospital, Seoul 06654, Republic of Korea; olinny87@hotmail.com (J.S.B.); missbunny0925@gmail.com (S.O.J.); spinesurgeryscope@hotmail.com (K.-H.S.)

**Keywords:** transforaminal lumbar interbody fusion, TLIF, unilateral instrumentation, bilateral instrumentation, coronal alignment, long-term outcome

## Abstract

*Background and Objective*: There is a paucity of literature comparing unilateral instrumented transforaminal lumbar interbody fusion (UITLIF) with bilateral instrumented TLIF (BITLIF) regarding radiological alignment, including the coronal balance, even though UITLIF might have asymmetric characteristics in the coronal plane. This retrospective study aimed to compare the clinical and long-term radiological outcomes of 1-level UITLIF and BITLIF in lumbar degenerative diseases (LDD) including lumbar spinal stenosis with or without spondylolisthesis (degenerative or spondylolytic). *Materials and Methods*: Patients who underwent 1-level UITLIF with two rectangular polyetheretherketone (PEEK) cages or BITLIF between November 2009 and June 2016 by four surgeons with ≥5 years of follow-up at a single hospital were included. We compared the clinical and radiological outcomes between the UITLIF and BITLIF. *Results*: In total, 63 and 111 patients who underwent UITLIF and BITLIF, respectively, were enrolled. The median follow-up was 85.55 months (range: 60–130). The UITLIF group had a significantly shorter operation time (185.0 [170.0–210.0] vs. 225.0 [200.0–265.0], *p* < 0.001) and lower estimated blood loss (300.0 [250.0–500.0] vs. 550.0 [400.0–800.0], *p* < 0.001) than the BITLIF group. Regarding the clinical outcomes, there were no significant differences in the intermittent claudication score (*p* = 0.495) and Kirkaldy–Willis criteria (*p* = 0.707) at 1 year postoperatively. The interval changes in the local coronal Cobb angle at the index level, L1-S1 lordotic angle, and coronal off-balance from the immediate postoperative radiograph to the last follow-up were not significantly different (*p* = 0.687, *p* = 0.701, and *p* = 0.367, respectively). *Conclusions*: UITLIF with two rectangular PEEK cages may provide comparable clinical outcomes and radiological longevity including coronal alignment to BITLIF in 1-level LDD. In addition, UITLIF has advantages over BITLIF in terms of operative time and blood loss.

## 1. Introduction

Lumbar fusion is an efficient surgical treatment for various lumbar degenerative diseases (LDD), including spinal stenosis, spondylolisthesis, and deformity [1,2,3]. Among several fusion techniques, transforaminal lumbar interbody fusion (TLIF), a modification of posterior lumbar interbody fusion (PLIF), has become a popular and effective procedure since its initial description in the 1990s [4,5]. TLIF is known to have some advantages, such as a smaller incision, shorter operation time, less blood loss, less neurological injury, and less destruction of posterior elements when compared to PLIF [2,6,7].

Bilateral instrumented TLIF (BITLIF) has been reported to provide clinical and radiological outcomes comparable to those of PLIF in previous studies [2,8]. Although BITLIF is generally considered a standard technique, unilateral instrumented TLIF (UITLIF) has also been reported to be as effective as BITLIF and shows better results, especially in terms of the operation time and blood loss in previous studies [5,9]. In contrast, several researchers have reported increased rates of pseudarthrosis and reoperations in UITLIF than those in BITLIF [10,11].

Since UITLIF has asymmetric characteristics in the coronal plane, including unilateral facet resection, muscle dissection, location of interbody implants, and screw fixation, it is reasonable to have doubt regarding the long-term outcomes. However, to our knowledge, little is known about the long-term radiological alignment outcomes of UITLIF since most of the previous studies related to TLIF have radiologically concentrated on the fusion rate, intervertebral disc height (IDH), or metal failure with relatively short-term follow-up [1,5,12,13]. In addition, coronal alignment has not been thoroughly addressed. Therefore, the purpose of the present study was to compare the clinical outcomes and radiological longevity of UITLIF and BITLIF in patients with LDD. Specifically, we aimed to evaluate the radiological results for fusion rates and metal failure, as well as the spinal alignment between the two surgical procedures. We hypothesized that UITLIF would have comparable radiological longevity, less intraoperative blood loss, and shorter duration of operation compared to BITLIF.

## 2. Materials and Methods

### 2.1. Patients

This retrospective study was approved by the institutional review board of the Ministry of Health and Welfare, and a waiver of consent was obtained. This study reviewed all consecutive patients who underwent 1-level UITLIF or BITLIF between November 2009 and June 2016 at a single institution and were followed up for more than 5 years. We included the patients who were treated for LDD: (1) degenerative spondylolisthesis; (2) spondylolytic spondylolisthesis; and (3) spinal stenosis without spondylolisthesis or instability. The exclusion criteria were as follows: (1) surgery for infection, trauma, or adjacent segment disease (ASD) or nonunion after previous lumbar fusion surgeries; (2) patients who were simultaneously diagnosed with LDD at another level requiring surgery soon; (3) the occurrence of vertebral fracture at other segments affecting radiological parameters during the follow-up period; or (4) incomplete medical record documentation.

### 2.2. Surgical Procedures

**Figure 1:**
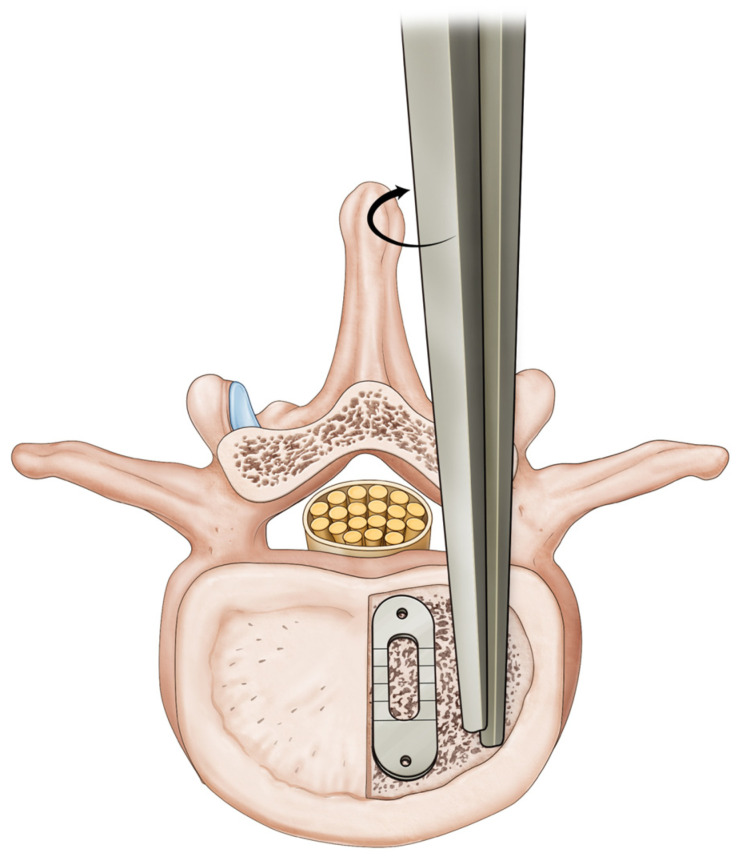
Technique for advancement of the first cage toward the midline.

Four surgeons from a single spine surgery center performed operations with the same surgical procedures. The approach side was determined based on the dominant side of the patient’s symptoms and preoperative radiological findings, including laterality of stenosis and/or closed side of disc wedging. The surgeons use a midline or paraspinal approach based on the location of the main pathology. For example, the paraspinal approach was preferred in patients with extraforaminal pathologies. Contralateral intracanal decompression was carried out through an “over-the-top” approach when the patient had central canal stenosis and concomitant contralateral symptoms. After laminectomy, facetectomy, and discectomy unilaterally, two polyetheretherketone (PEEK) cages packed with autogenous local bone chips and demineralized bone matrix were inserted into the intervertebral space. Swiveling a blunt chisel against the other within the disc space would move the first cage toward the midline to secure the stability and space for the second cage (Figure 1). The second cage is located on the apophyseal ring. The disc height was restored to the same extent as that of the contralateral side. All the procedures were performed under a microscope. Finally, unilateral or bilateral pedicle screw fixation (PSF) was performed. Contralateral PSF was conducted using a midline or paraspinal approach depending on the surgeon’s preference in BITLIF.

### 2.3. Data Collection and Outcome Assessment

**Figure 2:**
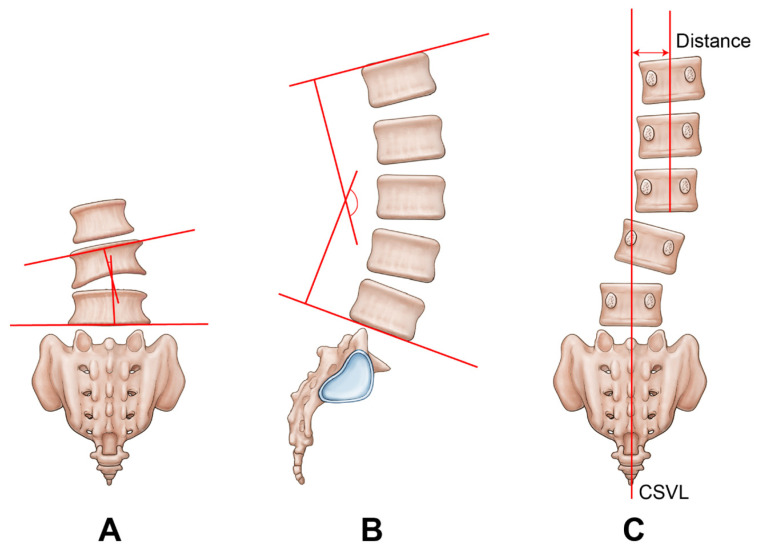
Measurements of radiological parameters. (**A**) Coronal Cobb angle at the index level (local coronal angle). (**B**) L1-S1 lordotic angle. (**C**) Lumbar coronal off balance. CSVL, central sacral vertical line.

Patients were followed up at 3, 6, and 12 months, as well as 2 and 5 years postoperatively. We collected data on patient demographics (sex and age), preoperative bone mineral density (BMD), and perioperative details (diagnosis, operated level/location, operation time, and intraoperative estimated blood loss (EBL)). The clinical outcomes were assessed based on the severity of intermittent claudication (Japanese Orthopedic Association score 0–3) preoperatively and at 1 year postoperatively and based on the Kirkaldy–Willis criteria at 1 year follow-up (Table 1) [14,15]. Radiological alignments were measured with the coronal Cobb angle at the index disc level (local coronal angle, LCA), L1-S1 lordotic angle (LA), and lumbar coronal off balance (COB) using standing neutral plain radiographs preoperatively, immediately postoperatively, and at the last follow-up (Figure 2) [16,17].

Cage subsidence was defined as endplate disruption and cage sinking into the adjacent vertebral bodies on the lateral plain radiograph during the follow-up period with no evidence of endplate violation on the immediate postoperative radiograph. Bone fusion was evaluated at 1 year postoperatively by a three-dimensional computed tomography (CT) scan or plain radiograph when there was no CT scan. Fusion was determined by continuous bony trabeculation between two adjacent vertebral bodies on a CT scan or static radiograph and/or segmental movement of the fused level less than 4° on flexion-extension radiographs [1,5,18]. ASD was diagnosed as clinical neurologic claudication accompanied by a diagnosis of spinal stenosis on MRI, development of spondylolisthesis > 4 mm, segmental kyphosis > 10°, a complete collapse of the disc space, or deterioration of the intervertebral disc with two or more grades of the Weiner classification at the adjacent level [19,20]. The radiological outcomes were measured by a radiologist who was blinded to the study design. To analyze the intra- and inter-observer reliability, radiological parameters, including LCA, LA, and COB, were measured for 20 randomly selected patients by the above-mentioned radiologist with a 4-week interval and another author who was a neurosurgeon.

### 2.4. Statistical Analysis

Continuous variables were compared using Student’s *t*-test (mean ± standard deviation) or Mann–Whitney test (median, interquartile range); additionally, categorical variables were compared using the χ^2^ test or Fisher’s exact test. Linear mixed models (LMMs) were used to investigate within-patient correlation and interaction with time, modeled as fixed effects. Statistical significance was set at *p* < 0.05. All statistical analyses were performed using SPSS Statistics, version 25.0 (IBM, Armonk, NY, USA).

## 3. Results

### 3.1. Patients and Characteristics

A total of 293 consecutive patients who underwent 1-level UITLIF or BITLIF with a minimum of 5 years of follow-up were identified. Patients with surgery for infection, trauma, ASD, or nonunion (*n* = 27), LDD at another level requiring surgery soon (*n* = 48), the occurrence of vertebral fracture during the follow-up period (*n* = 4), or incomplete documentation (*n* = 40) were excluded, leaving 174 patients. Among them, 63 and 111 patients underwent UITLIF and BITLIF, respectively. These included 113 women and 61 men, with a mean age of 63.0 ± 8.1. The operated segment was L2–3 in 3 (1.7%), L3–4 in 14 (8.0%), L4–5 in 117 (67.2%), and L5–S1 in 40 patients (23.0%). Preoperative diagnoses were degenerative spondylolisthesis in 91 (52.3%), spondylolytic spondylolisthesis in 25 (14.4%), and spinal stenosis without spondylolisthesis and dynamic instability in 58 patients (33.3%). The median follow-up was 85.6 months (range: 60–130 months).

### 3.2. Comparison of Characteristics and Perioperative Information between Two Groups

When the UITLIF (*n* = 63) and BITLIF (*n* = 111) groups were compared, there was no significant difference in BMD between the groups. Patients who underwent UITLIF had a significantly shorter operation time (185.0 [170.0–210.0] vs. 225.0 [200.0–265.0], *p* < 0.001) and less EBL (300.0 [250.0–500.0] vs. 550.0 [400.0–800.0], *p* < 0.001) compared to those who underwent BITLIF (Table 2).

### 3.3. Comparison of Clinical and Radiological Outcomes between Two Groups

Regarding the clinical outcomes, the intermittent claudication score was not significantly different between the two groups at 1 year postoperatively (*p* = 0.495), even though patients with UITLIF had a lower score than those with BITLIF preoperatively (*p* = 0.039). Comparisons of radiological parameters, including LCA, LA, and COB, showed no statistically significant differences preoperatively, immediately postoperatively, and at the last follow-up between the two groups. In addition, there was no significant difference in the interval changes in LCA, LA, and COB (*p* = 0.687, *p* = 0.701, and *p* = 0.367, respectively) (Table 3).

The LMM analysis for intermittent claudication indicated that the type of surgical procedure did not significantly affect the improvement in claudication over time (Table 4). The LMM analyses for interval changes in the radiological parameters showed that the type of surgical procedure had no significant impact on interval changes in LCA, COB, and LA (Table 5).

Comparisons of postoperative complications, such as cage subsidence, pseudarthrosis, metal failure, ASD, and postoperative infection, revealed no significant differences between the groups (Table 6).

### 3.4. Intra- and Inter-Observer Reliabilities

All intraclass correlation coefficient values for intra- and inter-observer reliability were higher than 0.8, indicating excellent reliability.

## 4. Discussion

UITLIF would have comparable outcomes and less operation time and blood loss compared to BITLIF [5,9]. However, unilateral PSF may exacerbate asymmetric features of TLIF, such as unilateral muscle dissection, facet resection, and discectomy, which can cause coronal malalignment in the long run. Nevertheless, previous studies comparing these two procedures reported fusion rates and metal failure with relatively short-term follow-up rather than the long-term coronal alignment [5,10]. Accordingly, it is reasonable to compare UITLIF with BITLIF in terms of the long-term radiological outcomes, especially coronal balance.

It has been suggested that unilateral PSF can not only reduce medical costs, operation time, blood loss, and risk of complications but also result in a reliable fusion rate compared to bilateral PSF in various lumbar fusion surgeries [21,22]. Although bilateral PSF has been known to provide more stability and stiffness than unilateral PSF in previous biomechanical studies [23,24], several authors have proposed that excessive rigid fixation may induce stress shielding and subsequent regional osteopenia [25,26]. Xue et al. reported no differences in the fusion rate and screw failure between UITLIF and BITLIF with a single rectangular cage [5]. Aoki et al. also reported that UITLIF with one cage showed no difference in the fusion rate even with BITLIF with two cages [14]. In addition, Chen et al. suggested that unilateral PSF with two cages inserted bilaterally in the disc space might offer comparable stability compared with bilateral PSF plus bilaterally inserted two interbody cages in biomechanical testing [23]. They suggested that the anterior support of interbody cages could restore the spinal stability and torsional stiffness of unilateral PSF. Based on this idea, surgeons in our hospital have attempted to move the first cage to and beyond the midline to alleviate concerns about stability in UITLIF. The current study including 63 patients with UITLIF using two rectangular PEEK cages with a minimum of 5 years of follow-up showed a 95.2% fusion rate of UITLIF without a significant difference with BITLIF, consistent with the results of previous studies.

**Figure 3:**
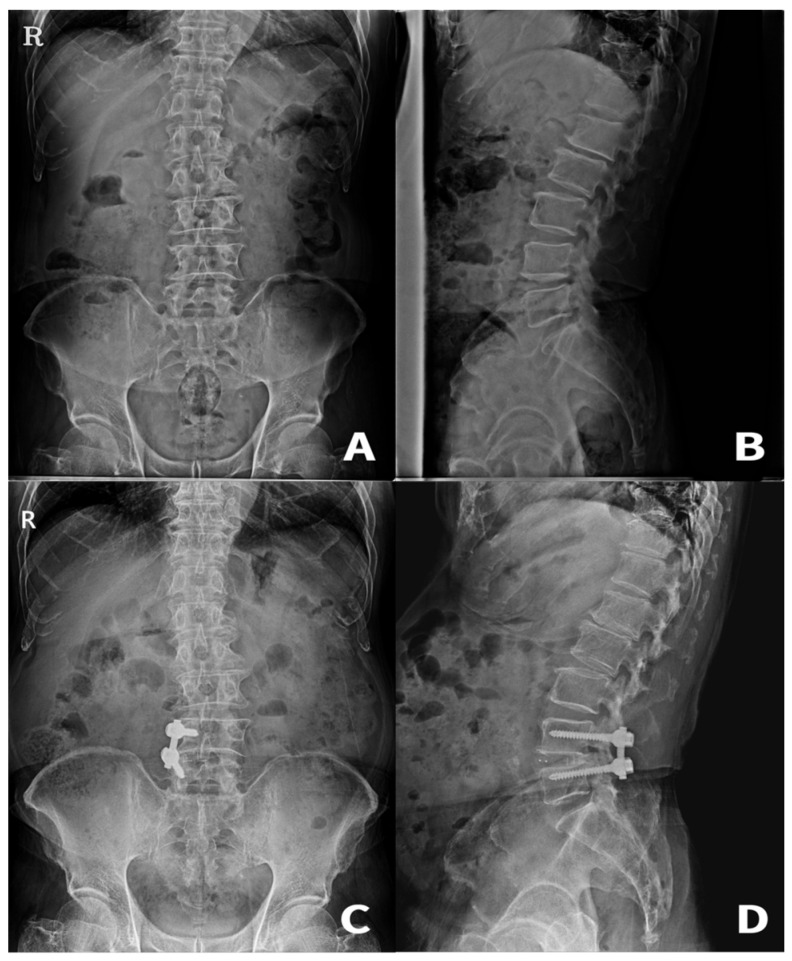
A 72-year-old male patient who underwent unilateral instrumented transforaminal lumbar interbody fusion for spinal stenosis combined with disc extrusion causing right-sided radiating pain unresponsive to conservative treatment. (**A**,**B**) Preoperative radiographs. (**C**,**D**) Radiographs at 65 months after surgery showing maintenance of the lumbar alignment.

There was no significant difference between the two groups with respect to the LCA, COB, and LA. Furthermore, LMMs with various covariates showed that the type of surgical procedure was not a significant factor in the maintenance of these parameters. This result indicates that UITLIF with two rectangular PEEK cages would not be inferior to BITLIF in the long run in terms of radiological parameters, including coronal alignment, despite the asymmetric nature of UITLIF in the coronal plane (Figure 3). To the best of our knowledge, the current study is the first to analyze the radiological parameters of a relatively large number of patients with UITLIF using two cages with a minimum of 5 years of follow-up.

Our results comparing UITLIF and BITLIF also show that both surgical procedures are beneficial in terms of intermittent claudication improvement and postoperative Kirkaldy–Willis criteria, which have been utilized to assess functional outcomes in various spinal diseases [13,27]. Although patients who underwent UITLIF had lower preoperative claudication scores than those who underwent BITLIF, UITLIF showed comparable postoperative clinical outcomes. However, several other widely used tools for clinical results, such as the Oswestry Disability Index and Short-Form Health Survey, were not documented in our retrospective cohort. In addition, while Lee et al. reported that TLIF showed better results on the immediate postoperative pain scale [1], it was impossible to evaluate the immediate postoperative pain in the current study due to a lack of consistent records. A well-designed prospective study evaluating the clinical outcomes of UITLIF and BITLIF is required in the future.

The ratio of preoperative diagnoses was different between the two groups in our cohort due to its retrospective study design. The LMMs analyses with various potential covariates including preoperative diagnoses were performed to address the effects of the different ratio of preoperative diagnoses. The results of LMMs revealed that preoperative diagnoses did not have a significant impact on radiological alignment. However, the different ratio of preoperative diagnoses between groups due to the retrospective study design might have limited the comparability of radiological parameters, since preoperative spondylolisthesis with instability may differ with spinal stenosis without spondylolisthesis in terms of the stabilization of the fused segment postoperatively. Although previous studies comparing BITLIF and PLIF in degenerative or isthmic spondylolisthesis have demonstrated comparable results regarding the fusion rate, IDH, slippage, and segmental lordosis [8,13], there is a lack of literature concentrating on the long-term outcomes of UITLIF in patients with spondylolisthesis. Therefore, a well-controlled study should be conducted in the future for more clarity.

There was a discrepancy in age between the two groups in the study cohort. Since the selection of the procedure was based not only on the symptoms and radiological findings but also on the patient’s condition, including age or comorbidities, there has been a tendency to perform UITLIF for elderly patients rather than BITLIF to reduce the burden of surgery. Moreover, there was no significant difference in the outcomes between the two groups, even though the patients in the UITLIF group were older than those in the BITLIF group. Nevertheless, this was a limitation of the retrospective study design.

We included patients who underwent 1-level UITLIF or BITLIF to enhance the homogeneity of the study cohort. Kai et al. documented that a 2-level UITLIF for LDD would have comparable results with BITLIF, with a reduced operation time and blood loss [22]. However, fusion status, screw failure, and other complications were assessed, excluding spinal alignment, with an unspecified follow-up period in that study. In addition, they reported a patient with proximal scoliosis 3 months after 2-level UITLIF. Thus, further studies are needed to evaluate the outcomes of multilevel UITLIF.

Although we suggested UITLIF with two rectangular PEEK cages as an alternative option for LDD, its role may be still limited to 1-level LDD. Additionally, the effectiveness of UITLIF in patients with severe instability has not been clarified despite the statistical supplementation for the different ratio of preoperative diagnoses between groups. Therefore, we suggest that UITLIF should be considered with meticulously careful attention for the strictly selected patients.

The current study has several limitations. First, owing to its retrospective nature, there may be selection bias and confounding factors that have not been considered. Specifically, we could not evaluate patients who did not visit our hospital more than 5 years after surgery, which might have influenced our results. Second, although smoking and obesity may be associated with unsuccessful fusion or postoperative complications after spinal fusion surgery, there were no consistent records of the smoking status or obesity in our retrospective cohort. Third, although the LMM analyses indicated no significant impact of different ratios of preoperative diagnoses between groups on the outcomes, it may limit the comparability. Finally, incomplete documentation of certain clinical data, such as pain intensity during the immediate postoperative and follow-up periods, could limit the interpretation of the results. Despite these limitations, this is the first study to compare the clinical and radiological outcomes between UITLIF and BITLIF and document the coronal alignment after UITLIF using two rectangular PEEK cages with a relatively large number of patients and a minimum of 5 years of follow-up.

## 5. Conclusions

UITLIF with two rectangular PEEK cages would not be inferior to BITLIF in both clinical outcomes and radiological longevity including coronal alignment in 1-level LDD. In addition, UITLIF has advantages over BITLIF in terms of the duration of the operation and EBL.

## Figures and Tables

**Table 1:** Assessment of intermittent claudication and Kirkaldy–Willis criteria for clinical outcomes.

	Score
Intermittent claudication	
Normal	3
Able to walk > 500 m but with pain, tingling, and/or muscle weakness	2
Able to walk > 500 m because of pain, tingling, and/or muscle weakness	1
Able to walk > 100 m because of pain, tingling, and/or muscle weakness	0
Kirkaldy–Willis criteria	
The patient has returned to their normal work and other activities with little or no complaints	Excellent
The patient has returned to their normal work but may have some restriction in other activities and may on occasion after heavy work have recurrent back pain requiring rest for a few days	Good
The patient has to reduce their working capacity, take a lighter job or work part-time, and occasionally have a recurrence of pain requiring absence from work for 1–2 weeks, once or twice a year	Fair
The patient does not return to work	Poor

**Table 2:** Comparison of baseline characteristics and perioperative information between patients with UITLIF and PLIF.

	UITLIF (*n* = 63)	BITLIF (*n* = 111)	*p*-Value
Age (yrs)	68 (62, 71)	61 (55, 68)	<0.001 *^,†^
Sex, *n* (%)			0.145 ^‡^
Female	36 (57.1)	77 (69.4)	
Male	27 (42.9)	34 (30.6)	
BMD, T-score	−2.66 (−3.41, −1.83)	−2.56 (−3.17, −1.59)	0.259 ^†^
Fused segment, *n* (%)			0.859 ^§^
L2-3	1 (1.6)	2 (1.8)	
L3-4	6 (9.5)	8 (7.2)	
L4-5	40 (63.5)	77 (69.4)	
L5-S1	16 (25.4)	24 (21.6)	
Diagnosis, *n* (%)			0.012 *^,‡^
Degenerative spondylolisthesis	17 (27.0)	74 (66.7)	
Spondylolytic spondylolisthesis	3 (4.8)	22 (19.8)	
Spinal stenosis without spondylolisthesis or instability	43 (68.3)	15 (13.5)	
Operation time (min)	185.0 (170.0, 210.0)	225.0 (200.0, 265.0)	<0.001 *^,†^
EBL (mL)	300.0 (250.0, 500.0)	550.0 (400.0, 800.0)	<0.001 *^,†^
Follow-up (months)	82.0 (66.0, 98.0)	85.0 (72.0, 85.0)	0.252 ^†^

The data are presented as the median (interquartile range) unless otherwise indicated. UITLIF, unilateral instrumented transforaminal lumbar interbody fusion; BITLIF, bilateral instrumented transforaminal lumbar interbody fusion; BMD, bone mineral density; EBL, estimated blood loss. * Statistically significant (*p* < 0.05). ^†^ Mann–Whitney test. ^‡^ Chi-squared test with continuity correction. ^§^ Fisher’s exact test.

**Table 3:** Comparison of radiological and clinical outcomes between patients with UITLIF and PLIF.

	UITLIF (*n* = 63)	BITLIF (*n* = 111)	*p*-Value
Radiological outcomes			
LCA (°)			
Preoperative	2.09 (5.53, 5.53)	1.38 (0.63, 3.87)	0.125 ^†^
Postoperative	1.03 (1.70, 1.70)	0.97 (0.60, 1.86)	0.344 ^†^
Last follow-up	1.74 (3.34, 3.34)	1.27 (0.64, 2.37)	0.201 ^†^
Interval change	0.73 (1.48, 1.48)	0.73 (0.39, 1.37)	0.687 ^†^
LA (°)			
Preoperative, mean ± SD	38.98 ± 12.88	42.41 ± 14.16	0.114 ^‡^
Postoperative, mean ± SD	41.67 ± 10.28	43.91 ± 10.79	0.183 ^‡^
Last follow-up, mean ± SD	38.48 ± 12.82	42.25 ± 13.6	0.075 ^‡^
Interval change	4.01 (6.97, 6.97)	3.93 (1.71, 7.74)	0.701 ^†^
COB (mm)			
Preoperative	5.98 (10.9, 10.9)	5.47 (2.96, 9.20)	0.823 ^†^
Postoperative	3.80 (6.97, 6.97)	4.72 (1.84, 8.10)	0.234 ^†^
Last follow-up	4.02 (8.04, 8.04)	4.94 (2.20, 8.81)	0.195 ^†^
Interval change	3.86 (6.52, 6.52)	4.54 (2.01, 6.86)	0.367 ^†^
Clinical outcomes			
Intermittent claudication scores			
Preoperative	1 (0, 1)	1 (0,1)	0.039 *^,†^
1 year postoperative	2 (2, 2)	2 (2, 2)	0.495^†^
K-W criteria 1 year postoperative, *n* (%)			0.707 ^§^
Excellent/Good	49 (77.8)	89 (80.2)	
Fair/Poor	14 (22.2)	22 (19.8)	

The data are presented as the median (interquartile range) unless otherwise indicated. UITLIF, unilateral instrumented transforaminal lumbar interbody fusion; BITLIF, bilateral instrumented transforaminal lumbar interbody fusion; LCA, local coronal angle; LA, L1-S1 lordotic angle; SD, standard deviation; COB, coronal off balance; K-W, Kirkaldy–Willis. * Statistically significant (*p* < 0.05). ^†^ Mann–Whitney test. ^‡^ Student’s *t*-test. ^§^ Chi-squared test with continuity correction.

**Table 4:** Linear mixed model for intermittent claudication scores (Type 3 tests of fixed effects).

Effect	Num DF	Den DF	F Value	*p*-Value
Operation type	1	172	3.37	0.068
Time	1	172	514.7	<0.001 *
Operation type × Time	1	172	2.18	0.1415

* Statistically significant (*p* < 0.05).

**Table 5:** Linear mixed models for interval changes in radiological parameters (Type 3 tests of fixed effects).

Variable	Effect	Num DF	Den DF	F Value	*p*-Value
LCA	Operation type	1	157	0.187	0.666
	Preoperative LCA	1	157	1.089	0.298
	Postoperative LCA	1	157	2.124	0.147
	Sex	1	157	0.661	0.417
	Age	1	157	0.021	0.884
	Surgeon	4	157	1.293	0.275
	Fused segment	3	157	1.434	0.235
	Subsidence	1	157	0.027	0.870
	Diagnosis	2	157	0.478	0.621
	BMD	1	157	0.000	0.988
COB	Operation type	1	157	0.958	0.329
	Preoperative COB	1	157	<0.001	0.992
	Postoperative COB	1	157	18.586	<0.001 *
	Sex	1	157	0.002	0.966
	Age	3	157	1.897	0.170
	Surgeon	4	157	0.143	0.966
	Fused segment	3	157	0.950	0.418
	Subsidence	1	157	2.972	0.087
	Diagnosis	2	157	0.133	0.875
	BMD	1	157	1.461	0.229
LA	Operation type	1	157	0.339	0.561
	Preoperative LA	1	157	3.965	0.048 *
	Postoperative LA	1	157	0.983	0.323
	Sex	1	157	0.958	0.329
	Age	1	157	0.027	0.870
	Surgeon	4	157	0.857	0.491
	Fused segment	3	157	0.666	0.574
	Subsidence	1	157	1.690	0.196
	Diagnosis	2	157	1.108	0.333
	BMD	1	157	0.793	0.375

LCA, local coronal angle; BMD, bone mineral density; COB, coronal off balance; LA, lordotic angle. Operation type, preoperative and postoperative parameters, sex, age, surgeon, fused segment, subsidence, diagnosis, and BMD were included in the linear mixed models. * Statistically significant (*p* < 0.05).

**Table 6:** Comparison of postoperative complications between patients with UITLIF and PLIF.

	UITLIF (*n* = 63)	BITLIF (*n* = 111)	*p*-Value
Cage subsidence, *n* (%)	8 (12.7)	8 (7.2)	0.351 *
Pseudarthrosis, *n* (%)	3 (4.8)	2 (1.8)	0.354 ^†^
Metal failure, *n* (%)	2 (3.2)	4 (3.6)	1.000 ^†^
ASD, *n* (%)	34 (54.0)	58 (52.3)	0.952 *
Postoperative infection, *n* (%)	2 (3.2)	5 (4.5)	1.000 ^†^

UITLIF, unilateral instrumented transforaminal lumbar interbody fusion; BITLIF, bilateral instrumented transforaminal lumbar interbody fusion; ASD, adjacent segment disease. * Chi-squared test with continuity correction. ^†^ Fisher’s exact test.

## Data Availability

The data presented in this study are available upon reasonable request from the corresponding author.
